# Supplementary material for: Medial preoptic area FoxO1 controls metabolic adaptation in a sexually dimorphic manner
Source: bioRxiv. 2025 Jun 28:2025.06.25.661575. Preprint. [Version 1] doi: 10.1101/2025.06.25.661575 (PMC12262617; doi:10.1101/2025.06.25.661575)
Supplement: 1 [file NIHPP2025.06.25.661575V1-supplement-1.pdf]

bioRxiv preprint doi: https://doi.org/10.1101/2025.06.25.661575; this version posted June 28, 2025. The copyright holder for this preprint (which was not certified by peer review) is the author/funder, who has granted bioRxiv a license to display the preprint in perpetuity. It is made available under aCC-BY 4.0 International license.

Figure S1

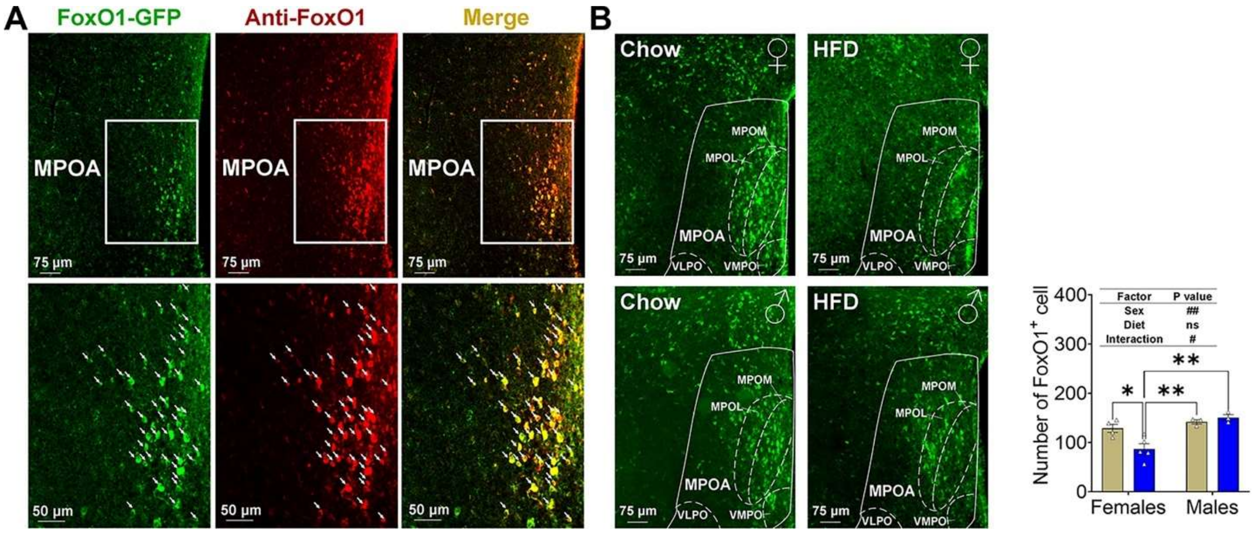

Figure S1. Sex-specific FoxO1 response to diet in the MPOA detected by FoxO1 immunostaining.

(A) Representative images showing the colocalization of FoxO1-GFP and FoxO1 immunofluorescence signals using anti-FoxO1 antibody. White arrowheads point to neurons expressing both GFP and FoxO1.

(B) FoxO1 immunofluorescence and quantification in the MPOA of female or male C57BL/6J mice (8 weeks) after two weeks of either chow diet or HFD feeding are shown.

Results are expressed as means ± SEM. (B), #$P < 0.05$, ##$P < 0.01$ in the two-way ANOVA analysis, *$P < 0.05$, **$P < 0.01$ in the subsequent post hoc Sidak tests.

bioRxiv preprint doi: https://doi.org/10.1101/2025.06.25.661575; this version posted June 28, 2025. The copyright holder for this preprint (which was not certified by peer review) is the author/funder, who has granted bioRxiv a license to display the preprint in perpetuity. It is made available under aCC-BY 4.0 International license.

Figure S2

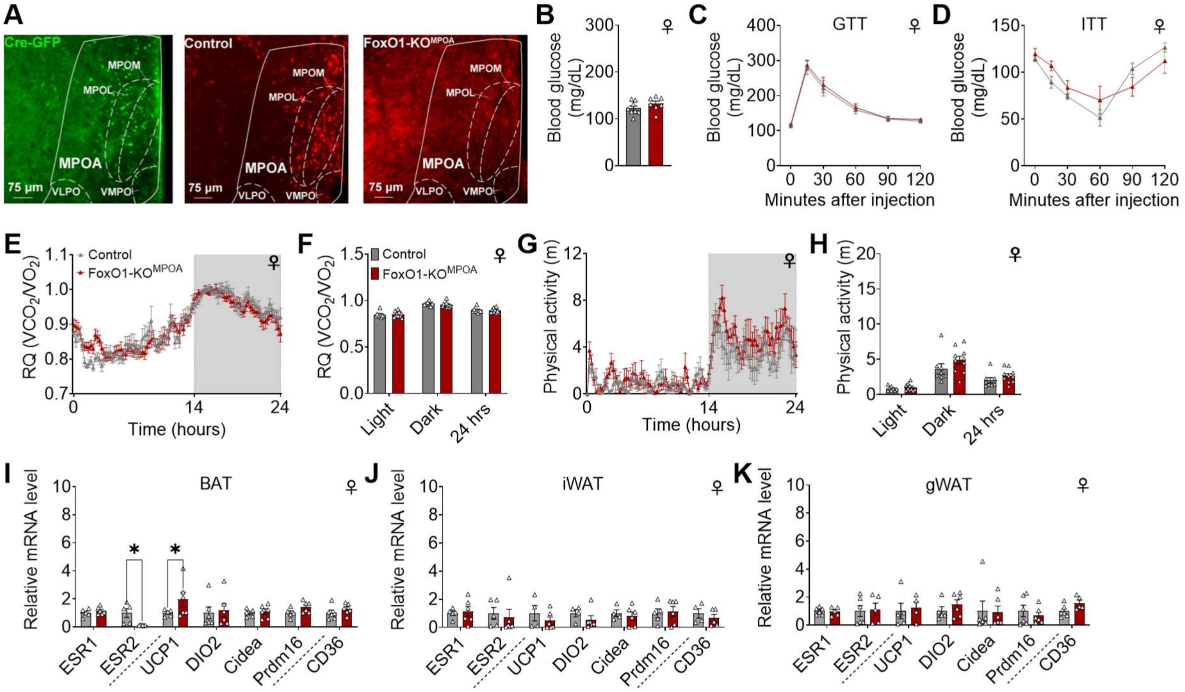

Figure S2. Effect of FoxO1-KO^{MPOA} on female metabolic parameters

(A) GFP immunoreactivity (left), FoxO1 immunoreactivity in control (middle), and FoxO1-KO^{MPOA} mice. AAV-CMV-Cre-GFP was injected into the MPOA of FoxO1$^{flox/flox}$ mice to generate FoxO1-KO^{MPOA} mice. Control mice were FoxO1$^{flox/flox}$ mice receiving AAV-CMV-GFP virus injections.

(B) Fed blood glucose levels measured 25 weeks after virus injection (n = 8/7).

(C-D) Glucose tolerance tests (C) and insulin tolerance tests (D) were performed 7 and 8 weeks after the virus injection (n = 8/8), using a separate cohort from the body weight recording group.

(E-H) RQ (E), light/dark/24-hour average RQ (F), physical activity (G), and light/dark/24-hour average physical activity (H) in female mice (n = 7/8).

(I-K) mRNA levels of genes in BAT (I), iWAT (J), and gWAT (K) of female mice 25 weeks after virus injections. Measured genes include estrogen receptors (ESR1 and ESR2), thermogenic genes (UCP1, Dio2, Cidea, PRDM16), and a fatty acid sensor and transporter (CD36, n = 6/6).

Results are shown as means ± SEM.

(I) *$P < 0.05$ in unpaired *t* tests.

bioRxiv preprint doi: https://doi.org/10.1101/2025.06.25.661575; this version posted June 28, 2025. The copyright holder for this preprint (which was not certified by peer review) is the author/funder, who has granted bioRxiv a license to display the preprint in perpetuity. It is made available under aCC-BY 4.0 International license.

Figure S3

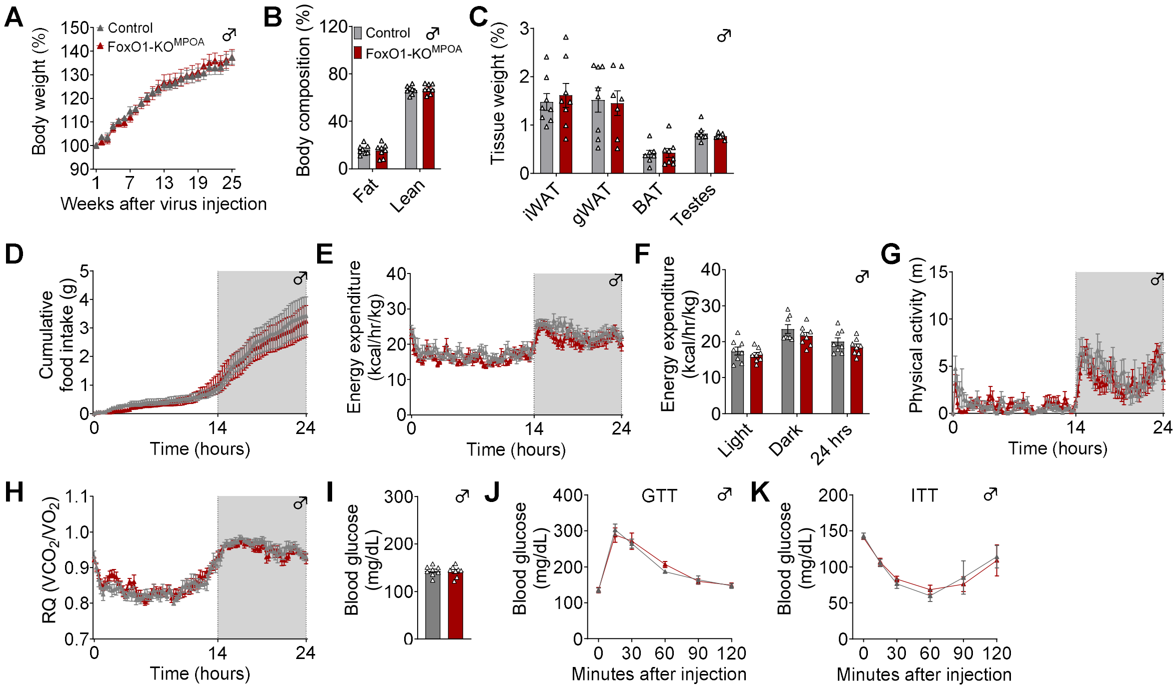

Figure S3. Glucose and energy homeostasis unaffected by FoxO1-KO^{MPOA} in male mice

(A) Body weight percentage compared to baseline one week after surgery. Male control and FoxO1-KO^{MPOA} mice received virus injections at 8 weeks of age and were fed a chow diet for 25 weeks (n = 8/8).

(B-C) Body composition as percentage of total body weight (B) and tissue index as percentage of body weight (C) in male mice 25 weeks after virus injection (n = 8/8).

(D-H) Cumulative food intake (D), energy expenditure (E), average energy expenditure during light/dark cycles and over 24 hours (F), physical activity (G), and RQ (H) in male mice (n = 8/8).

(I) Fed blood glucose levels measured 25 weeks after virus injection (n = 8/8).

(J-K) Glucose tolerance tests (J) and insulin tolerance tests (K) performed at 7 and 8 weeks after virus injection (n = 8/8). These tests were conducted in a separate cohort from the body weight measurements.

Results are presented as means ± SEM.

bioRxiv preprint doi: https://doi.org/10.1101/2025.06.25.661575; this version posted June 28, 2025. The copyright holder for this preprint (which was not certified by peer review) is the author/funder, who has granted bioRxiv a license to display the preprint in perpetuity. It is made available under aCC-BY 4.0 International license.

Figure S4

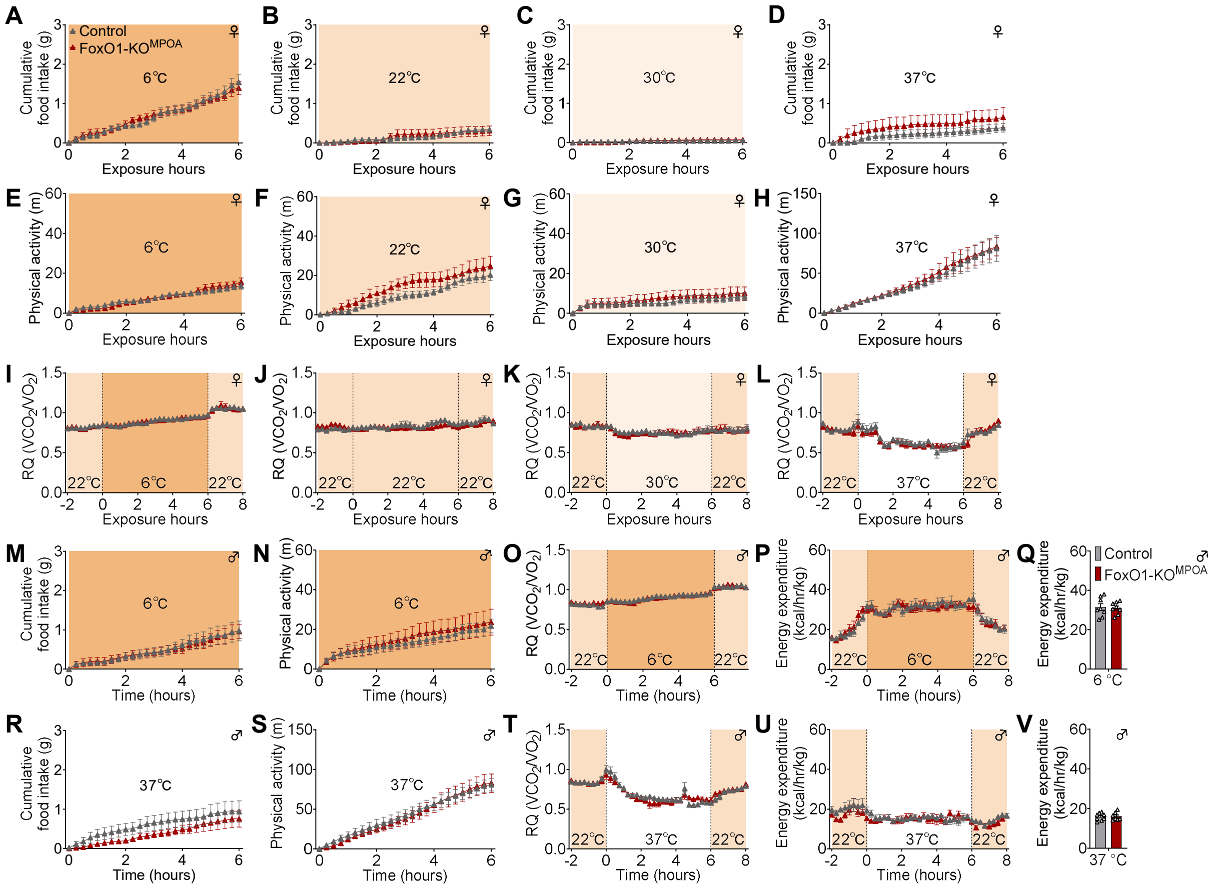

Figure S4. Effects of FoxO1-KO^{MPOA} on metabolic parameters during acute temperature challenges in male and female mice

(A–D) Cumulative food intake of female mice during 6-hour temperature exposures at 6°C (A, n = 9/8), 22°C (B, n = 5/7), 30°C (C, n = 9/10), and 37°C (D, n = 9/10).

(E–H) Physical activity of female mice during 6-hour temperature exposures at 6°C (E, n = 9/8), 22°C (F, n = 5/7), 30°C (G, n = 9/10), and 37°C (H, n = 9/10).

(I–L) RQ of female mice during 6-hour temperature exposures at 6°C (I, n = 9/8), 22°C (J, n = 5/7), 30°C (K, n = 9/10), and 37°C (L, n = 9/10).

(M–Q) Cumulative food intake (M), physical activity (N), RQ (O), energy expenditure (P), and average energy expenditure (Q) of male mice during 6-hour temperature exposure at 6°C (n = 8/9).

(R–V) Cumulative food intake (R), physical activity (S), RQ (T), energy expenditure (U), and average energy expenditure (V) of male mice during 6-hour temperature exposure at 37°C (n = 9/10).

Results are displayed as means ± SEM.

bioRxiv preprint doi: https://doi.org/10.1101/2025.06.25.661575; this version posted June 28, 2025. The copyright holder for this preprint (which was not certified by peer review) is the author/funder, who has granted bioRxiv a license to display the preprint in perpetuity. It is made available under aCC-BY 4.0 International license.

Figure S5

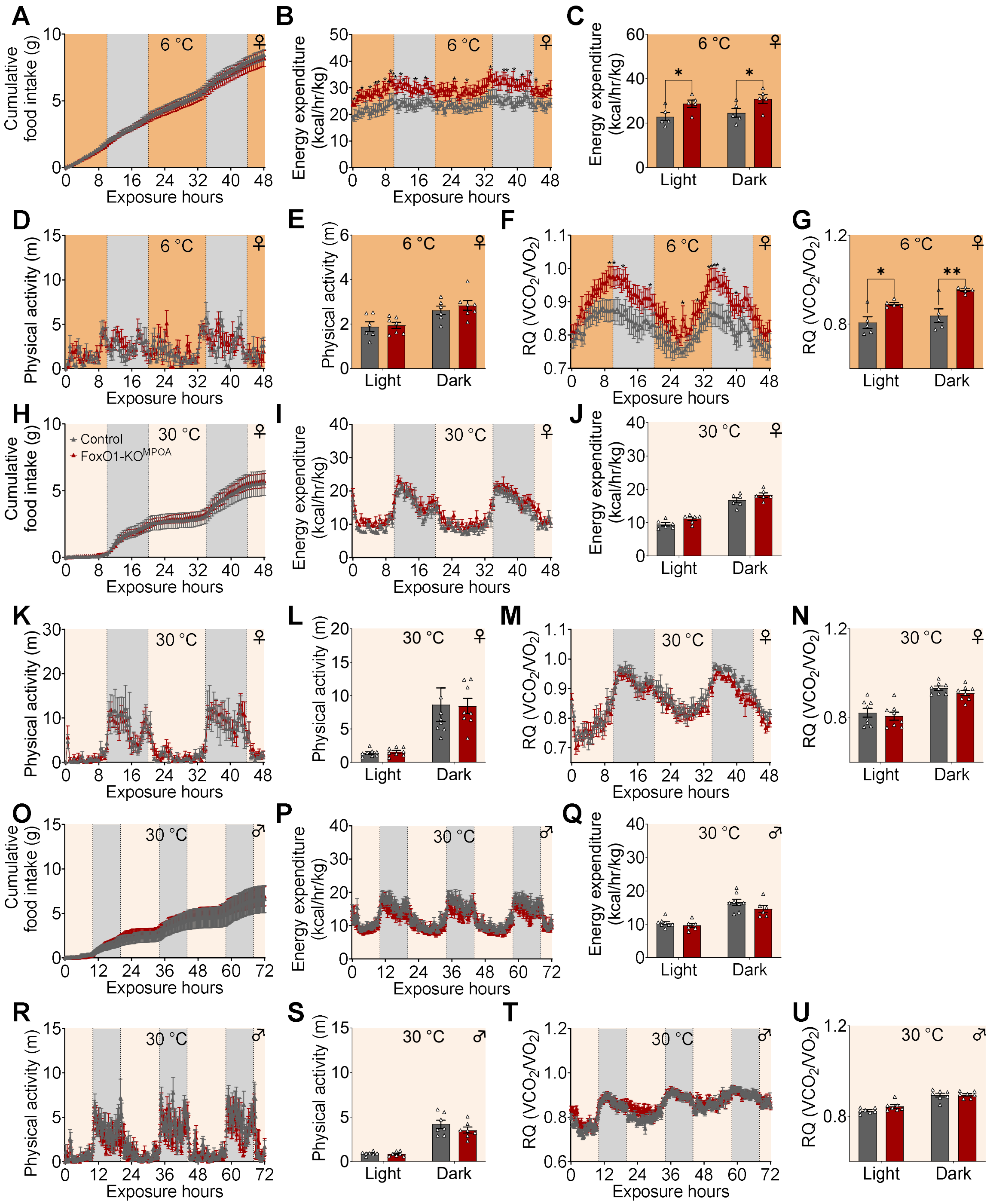

Figure S5. Effects of FoxO1-KO^{MPOA} on energy metabolism during chronic thermoneutral and cold Exposure

(A-G) Female mice during 48-hour cold exposure were analyzed for cumulative food intake (A, n = 7/8), energy expenditure (B, n = 5/5), light/dark average energy expenditure (C, n = 5/5), 30-

bioRxiv preprint doi: https://doi.org/10.1101/2025.06.25.661575; this version posted June 28, 2025. The copyright holder for this preprint (which was not certified by peer review) is the author/funder, who has granted bioRxiv a license to display the preprint in perpetuity. It is made available under aCC-BY 4.0 International license.

minute physical activity (D, n = 6/7), light/dark average physical activity (E, n = 6/7), RQ (F, n = 5/5), and light/dark average RQ (G, n = 5/5).

(H-N) Female mice during 48-hour thermoneutral exposure (n = 7/8) were measured for cumulative food intake (H), energy expenditure (I), light/dark average energy expenditure (J), 30-minute physical activity (K), light/dark average physical activity (L), RQ (M), and light/dark average RQ (N).

(O-U) Male mice during 72-hour thermoneutral exposure (n = 7/7) were assessed for cumulative food intake (O), energy expenditure (P), light/dark average energy expenditure (Q), 30-minute physical activity (R), light/dark average physical activity (S), RQ (T), and light/dark average RQ (U).

All measurements were conducted 8 weeks post-virus injection in mice with comparable body weight and lean mass. Data are presented as means ± SEM.

Statistical significance was determined by two-way ANOVA with post hoc Sidak tests for panels B-C and F-G (*$P < 0.05$, **$P < 0.01$).

bioRxiv preprint doi: https://doi.org/10.1101/2025.06.25.661575; this version posted June 28, 2025. The copyright holder for this preprint (which was not certified by peer review) is the author/funder, who has granted bioRxiv a license to display the preprint in perpetuity. It is made available under aCC-BY 4.0 International license.

Figure S6

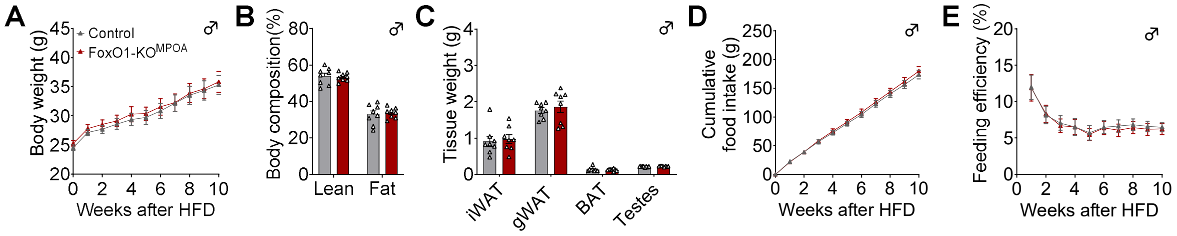

Figure S6. Energy homeostasis unchanged in male FoxO1-KO^{MPOA} mice on HFD

(A-C) Male mice were fed HFD for 10 weeks (n = 8 per group). Measurements show body weight (A), body composition as percentage of total weight (B), and tissue weights (C).

(D-E) Food intake and feeding efficiency in male control and FoxO1-KO^{MPOA} mice (n = 10 per group). Cumulative food intake (D) and feeding efficiency (E) were monitored during 10 weeks of HFD feeding, initiated 2 weeks post-virus injection at 10 weeks of age. Feeding efficiency represents the ratio of body weight change to cumulative food intake.

Data are presented as means ± SEM.

bioRxiv preprint doi: https://doi.org/10.1101/2025.06.25.661575; this version posted June 28, 2025. The copyright holder for this preprint (which was not certified by peer review) is the author/funder, who has granted bioRxiv a license to display the preprint in perpetuity. It is made available under aCC-BY 4.0 International license.

Figure S7

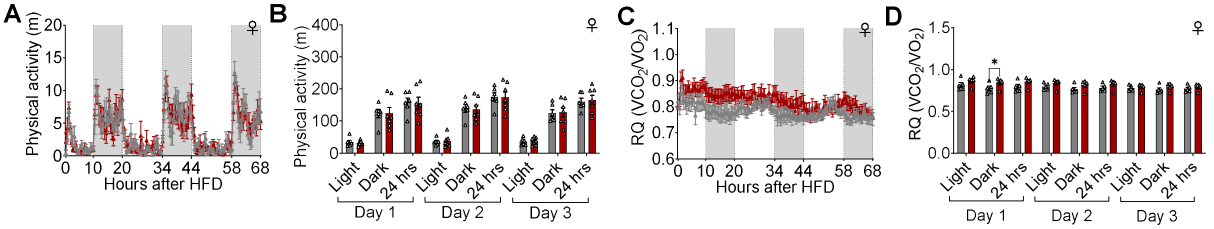

Figure S7. Physical activity and RQ remain unchanged in female FoxO1-KO^{MPOA} mice on HFD

(A-B) Physical activity measurements were recorded (A) and averaged across light/dark cycles and 24-hour periods (B, n = 6-7 mice per group).

(C-D) Respiratory quotient (RQ) was measured continuously (C) and averaged across light/dark cycles and 24-hour periods (D). Female control and FoxO1-KO^{MPOA} mice were acclimated to metabolic chambers for 2 days on chow diet before transitioning to HFD (n = 5 mice per group).

Data are presented as mean ± SEM.

(D) **P* < 0.05, two-way ANOVA with Sidak's post hoc test.

bioRxiv preprint doi: https://doi.org/10.1101/2025.06.25.661575; this version posted June 28, 2025. The copyright holder for this preprint (which was not certified by peer review) is the author/funder, who has granted bioRxiv a license to display the preprint in perpetuity. It is made available under aCC-BY 4.0 International license.

Figure S8

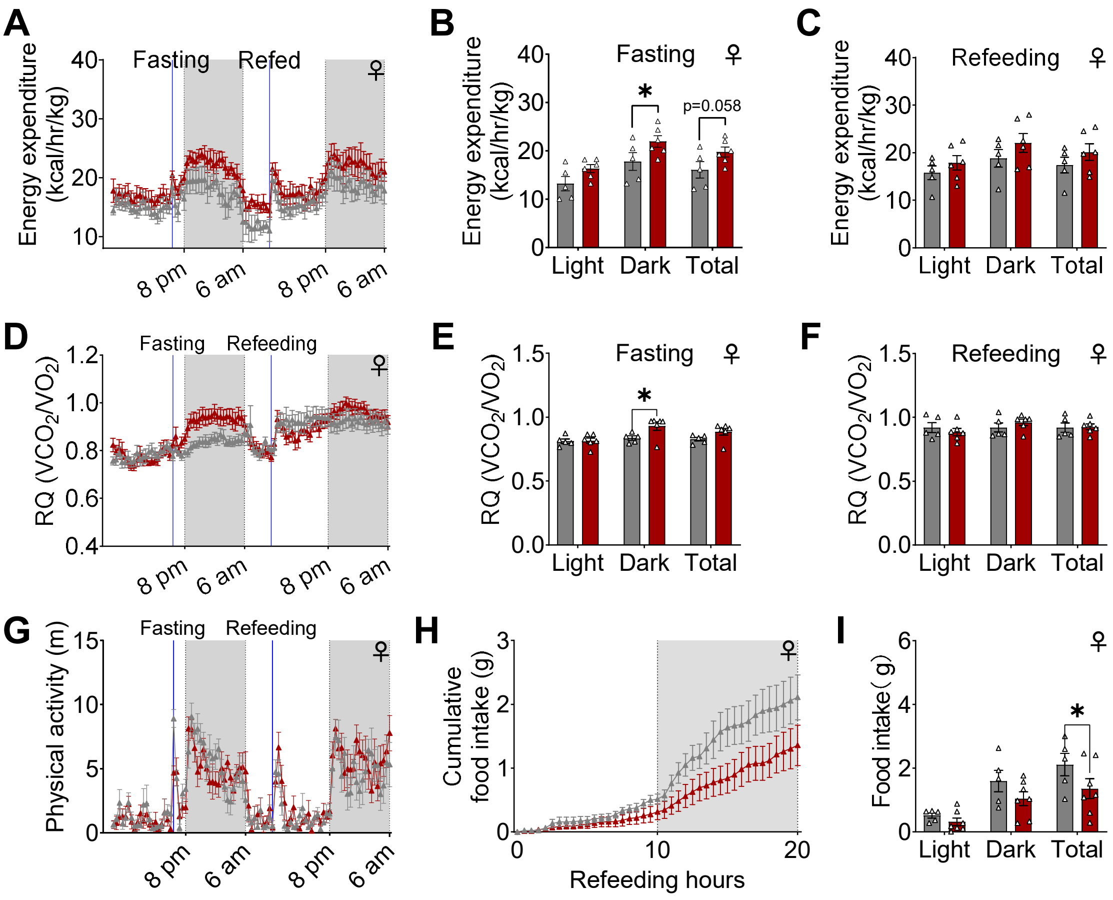

Figure S8. FoxO1-KO^{MPOA} increases energy expenditure during fasting and reduces fast-induced refeeding in female mice.

(A-I) Female control and FoxO1-KO^{MPOA} mice were acclimated to Sable Promethion System for 2 days with chow diet access, followed by overnight fasting and subsequent refeeding with chow diet (n=5-6 per group). (A) Time course of energy expenditure. (B,C) Average energy expenditure during (B) fasting and (C) refeeding periods. (D-F) RQ measurements (D) over time with averages during (E) fasting and (F) refeeding. (G) Physical activity levels. (H,I) Food intake shown as (H) cumulative intake and (I) total intake during refeeding period.

Data shown as mean ± SEM.

(B, E, I) *P < 0.05, two-way ANOVA with Sidak's post-hoc test.

bioRxiv preprint doi: https://doi.org/10.1101/2025.06.25.661575; this version posted June 28, 2025. The copyright holder for this preprint (which was not certified by peer review) is the author/funder, who has granted bioRxiv a license to display the preprint in perpetuity. It is made available under aCC-BY 4.0 International license.

Figure S9

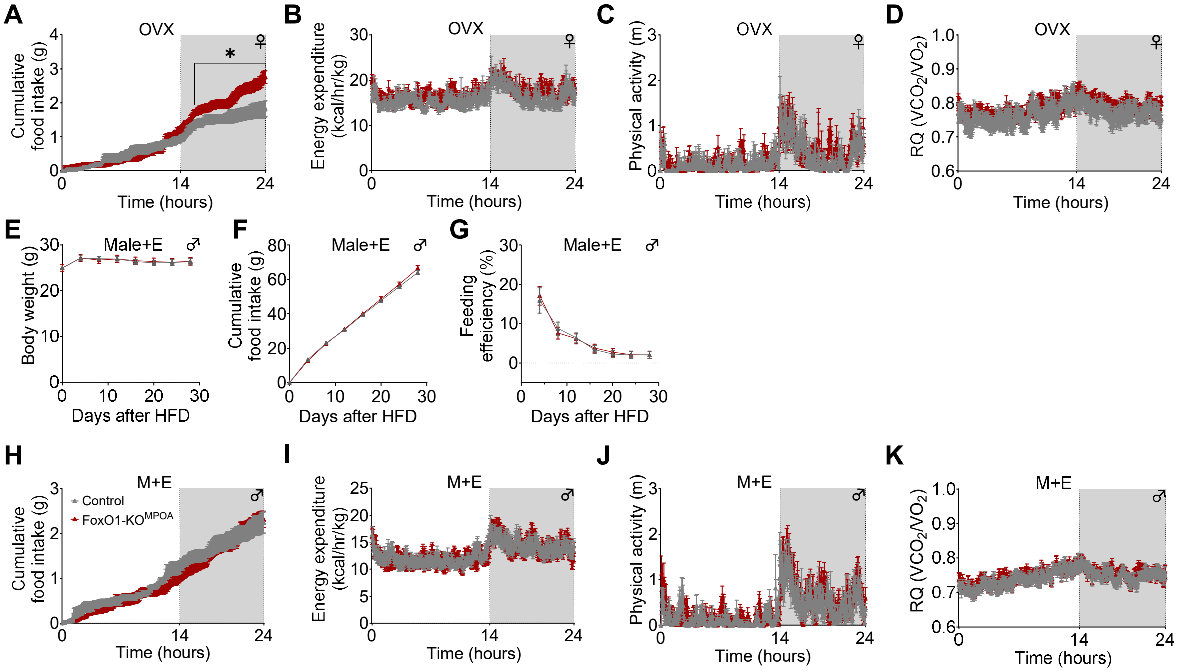

Figure S9. Effects of ovariectomy and 17β-estradiol supplementation on the FoxO1-KO^{MPOA}'s impact on energy homeostasis in mice

(A-D) Analysis of ovariectomized female control and FoxO1-KO^{MPOA} mice (n = 6-8) measuring cumulative food intake (A), energy expenditure (B), physical activity (C), and RQ (D).

(E-G) Male mice were implanted subcutaneously with 17β-estradiol pellets (Male+E, 0.025 mg/pellet, 60-day release, n = 8-9) and monitored for body weight (E), cumulative food intake (F), and feeding efficiency (G). HFD feeding began 4 days post-virus injection and E pellet implantation at 8 weeks of age.

(H-K) Analysis of E pellet-implanted male mice (Male+E, n = 8-9) measuring cumulative food intake (H), energy expenditure (I), physical activity (J), and RQ (K).

Mice were fed HFD starting 4 days after virus injection with concurrent OVX or E pellet implantation at 8 weeks of age. Metabolic measurements were conducted using the Sable Promethion System after 30 days on HFD.

Data represent means ± SEM.

(D) *P < 0.05 by two-way ANOVA with post hoc Sidak tests.

bioRxiv preprint doi: https://doi.org/10.1101/2025.06.25.661575; this version posted June 28, 2025. The copyright holder for this preprint (which was not certified by peer review) is the author/funder, who has granted bioRxiv a license to display the preprint in perpetuity. It is made available under aCC-BY 4.0 International license.

Figure S10

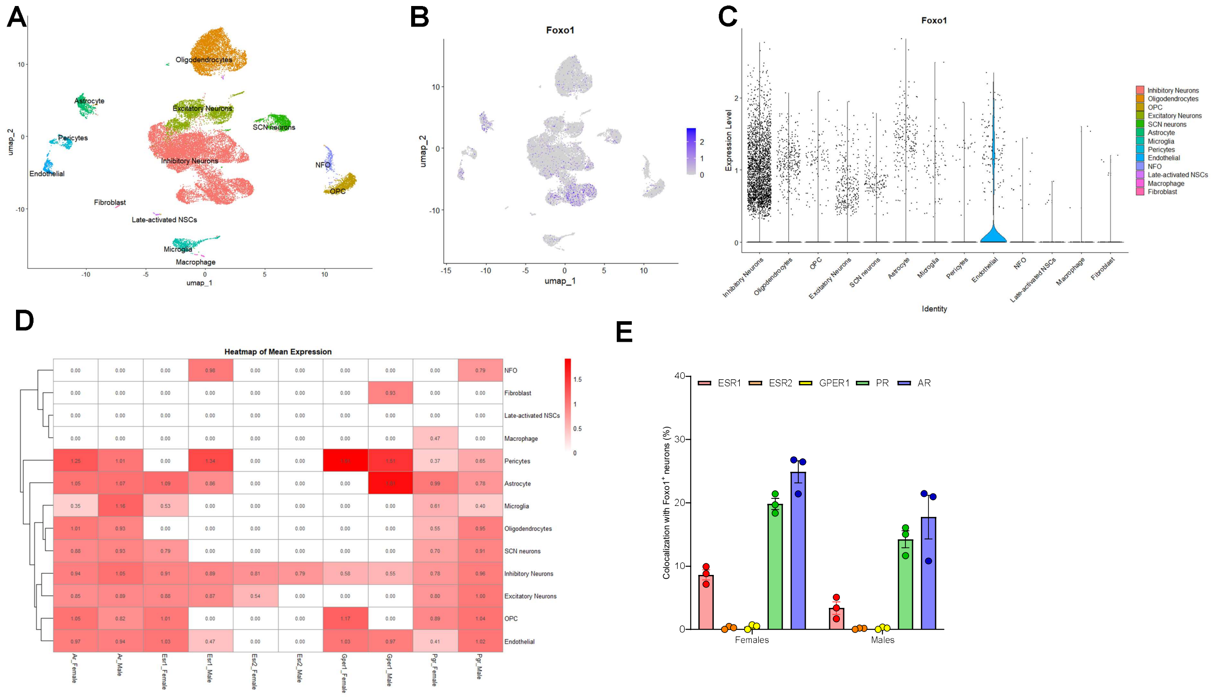

Figure S10. Cell type-specific expression and hormone receptor colocalization of FoxO1 in the MPOA

(A) t-distributed stochastic neighbor embedding (tSNE) visualization of MPOA cell clusters identified and annotated using SingleR and Azimuth algorithms. OPC: oligodendrocyte progenitor cells; SCN: Suprachiasmatic nucleus neurons; NFO: newly formed oligodendrocytes; NSCs: neural stem cells.

(B-C) Distribution of FoxO1 expression across identified cell populations shown as feature plot (B) and violin plot (C).

(D) Sex hormone receptor expression in FoxO1+ Cells. ESR1, estrogen receptor 1; ESR2, estrogen receptor 2; GPER1, G protein-coupled estrogen receptor 1; PR, progesterone receptor; AR, androgen receptor.

(E) Percentage of FoxO1+ inhibitory neurons expressing individual sex hormone receptor genes.

Data represent means ± SEM.
